# Maintenance and turnover of Sox2^+^ adult stem cells in the gustatory epithelium

**DOI:** 10.1371/journal.pone.0267683

**Published:** 2022-09-02

**Authors:** Makoto Ohmoto, Shugo Nakamura, Hong Wang, Peihua Jiang, Junji Hirota, Ichiro Matsumoto

**Affiliations:** 1 Monell Chemical Senses Center, Philadelphia, Pennsylvania, United States of America; 2 Department of Life Science and Technology, Graduate School of Life Science and Technology, Tokyo Institute of Technology, Yokohama, Kanagawa, Japan; 3 Faculty of Information Networking for Innovation and Design (INIAD), Toyo University, Kita, Tokyo, Japan; The University of Tokyo, JAPAN

## Abstract

Continuous turnover of taste bud cells in the oral cavity underlies the homeostasis of taste tissues. Previous studies have demonstrated that Sox2^+^ stem cells give rise to all types of epithelial cells including taste bud cells and non-gustatory epithelial cells in the oral epithelium, and Sox2 is required for generating taste bud cells. Here, we show the dynamism of single stem cells through multicolor lineage tracing analyses in *Sox2-CreERT2*; *Rosa26-Confetti* mice. In the non-gustatory epithelium, unicolored areas populated by a cluster of cells expressing the same fluorescent protein grew over time, while epithelial cells were randomly labeled with multiple fluorescent proteins by short-term tracing. Similar phenomena were observed in gustatory epithelia. These results suggest that the Sox2^+^ stem cell population is maintained by balancing the increase of certain stem cells with the reduction of the others. In the gustatory epithelia, many single taste buds contained cells labeled with different fluorescent proteins, indicating that a single taste bud is composed of cells derived from multiple Sox2^+^ stem cells. Our results reveal the characteristics of Sox2^+^ stem cells underlying the turnover of taste bud cells and the homeostasis of taste tissues.

## Introduction

Epithelial cells of the alimentary tract, including the oral cavity, are maintained by turnover in adult vertebrates. Multiple types of local resident stem cells continuously supply epithelial cells under normal conditions and replenish them after injury [1,2]. Lineage tracing with inducible Cre recombinase is a powerful tool for determining whether the cells expressing the gene of interest are stem cells [2]. This method has identified and distinguished the stem cells in the tongue epithelium that is maintained by continuous turnover and is composed of gustatory (i.e., taste bud) and non-gustatory epithelial cells. *Sox2*, *Krt5*, and *Krt14* are commonly expressed in tongue epithelial stem cells, which generate all types of epithelial cells in the tongue, including taste bud cells [3–5].

In the oral epithelium, epithelial stem cells can be categorized into two types: those that generate both taste bud cells and the epithelial cells surrounding taste buds in the gustatory papillae and those that generate only non-gustatory epithelial cells. *Lgr5* is expressed in stem cells distributed in the gustatory papillae located in the posterior part of the tongue, and *Lgr5*^+^ stem cells generate both taste bud cells and the surrounding epithelial cells in the papillae but not outside of it [6,7]. Because *Sox2* deficiency in epithelial stem cells leads to a marked decrease in *Lgr5* expression, *Lgr5*^+^ stem cells appear to be a subset of *Sox2*^+^*Krt5*^+^*Krt14*^+^ stem cells in the posterior gustatory papillae [4]. Like *Lgr5*^+^ stem cells, *Lgr6*^+^ stem cells give rise to a variety of taste bud cells; however, unlike *Lgr5*^+^ stem cells, they are distributed to the anterior gustatory papillae in addition to the posterior gustatory papillae [8]. It remains unclear whether *Lgr6*^+^ and *Lgr5*^+^ stem cells are identical or distinct in the posterior gustatory papillae. *Bmi1* is expressed in basal epithelial cells together with *Sox2*, *Krt5*, and *Krt14* in the oral epithelium, and *Bmi1*^+^ cells generate only non-gustatory epithelial cells [9,10]. Thus, oral epithelial stem cells can be characterized as either *Sox2*^+^*Bmi1*^*+*^*Krt5/14*^+^ non-gustatory epithelial stem cells or *Sox2*^+^*Lgr5-6*^*+*^*Krt5/14*^+^ gustatory epithelial stem cells expressing *Lgr5* and/or *Lgr6*.

Each taste bud is a cluster of tens of cells, distributed mainly in the soft palate, fungiform papillae scattered in the anterior two-thirds of the dorsal tongue, foliate papillae laterally located in the posterior tongue, and circumvallate papillae in the middle of the dorsal region of the posterior tongue. In mice, each fungiform papilla has a single taste bud, whereas foliate and circumvallate papillae contain many taste buds in the trench walls. Taste buds in the soft palate are buried in the epithelium without a discernible papillary structure. Single taste buds are composed of gustatory sensory cells called taste cells and putative non-sensory cells that are thought to provide structural support to the taste cells in the taste buds. Their average half-life is about 1–2 weeks [11,12], and thus they are continuously replaced by new cells from adult stem cells. In gustatory areas in the oral cavity, rapid-cycling stem cells that can be identified by the expression of a proliferation marker or the incorporation of thymidine analogs are *Sox2*^+^*Krt5/14*^+^, and actively generate post-mitotic precursor cells following terminal differentiation to all types of taste bud cells [3,13]. An *in vitro* organoid culture demonstrated that a single stem cell could generate multiple types of taste cell-like cells, with molecular features similar to those of taste cells and responding to taste stimuli, as well as non-sensory taste bud cells, suggesting that a single stem cell is capable of generating multiple types of cells in taste buds [8]. Although it is suggested that multiple local progenitor cells contribute to single taste buds in the circumvallate papillae [14,15], it remains unclear whether a single or multiple local resident stem cells maintain a taste bud in other gustatory areas *in vivo*.

In the present study, we characterized *Sox2*^+^*Bmi1*^*+*^*Krt5/14*^+^ non-gustatory and *Sox2*^+^*Lgr5-6*^*+*^*Krt5/14*^+^ gustatory epithelial stem cells by multicolor lineage tracing analyses in the oral epithelium using a confetti reporter strain. The number of filiform papillae contributing, at least in part, to a unicolored cluster in *Sox2*^*CreERT2/+*^*; Rosa26*^*Confetti/+*^ (*Sox2*-*Confetti*) mice increased significantly over time, indicating clonal expansion of *Sox2*^+^*Bmi1*^*+*^*Krt5/14*^+^ stem cells and supporting the previously reported population asymmetric division model with neutral competition [10]. Mathematical modeling, considering the composition and half-lives of individual cells in taste buds [12,16,17], revealed that almost all taste bud cells (>95%) were replaced with new cells within a few months; thus, we analyzed the distribution of fluorescent reporter expression in *Sox2*-*Confetti* mice with ≥4-month long chases. Many taste buds were composed of heterogeneous cells, as shown by the expression of fluorescent reporter proteins several months after the induction of reporter expression. The longer the chase, the more taste buds composed of homogeneously labeled cells were observed. These results suggest that cells in a single taste bud are derived from multiple *Sox2*^+^*Lgr5-6*^*+*^*Krt5/14*^+^ stem cells in all gustatory regions and that stem cells are maintained by the population asymmetric division model with neutral drift, similar to *Sox2*^+^*Bmi1*^*+*^*Krt5/14*^+^ non-gustatory stem cells [10] and intestinal stem cells [18,19].

## Materials and methods

### Animals

C57BL/6J (stock no. 000664), B6;129S-*Sox2*^*tm1(cre/ERT2)Hoch*^/J (*Sox2*^*CreERT2/+*^, stock no. 017593) [20], and B6.129P2-*Gt(ROSA)26Sor*^*tm1(CAG-Brainbow2*.*1)Cle*^/J (*Rosa26*^*Confetti/Confetti*^, stock no. 017492) [19] mice were purchased from the Jackson Laboratory. *Sox2*^*CreERT2/+*^; *Rosa26*^*Confetti/+*^ mice were obtained by mating *Sox2*^*CreERT2/+*^ mice and *Rosa26*^*Confetti/Confetti*^ mice. Mice from both sexes were used for analyses. All animal experiments were approved by the Institutional Animal Care and Use Committee of the Monell Chemical Senses Center and performed in accordance with the guidelines of National Institutes of Health.

### Tamoxifen administration

Tamoxifen (10 mg/ml in corn oil; Sigma-Aldrich) was intraperitoneally injected into 4–6 weeks old mice (100 mg/kg body weight) as previously described [3,4] once or daily for 5 days.

### Tissue preparation

*Sox2*^*CreERT2/+*^; *Rosa26*^*Confetti/+*^ mice were sacrificed at 3 days, 2 and 3 weeks, and 1, 2, 3, 4, 6, and 12 months after tamoxifen administration. Mice were deeply anesthetized with urethane and transcardially perfused first with PBS, then with 4% paraformaldehyde in PBS. Oral epithelia were dissected, post-fixed, cryoprotected, and frozen as described previously [3,4,21]. Cryosections of 8 μm thickness were cut using a Leica CM1900 cryostat (Leica Microsystems), mounted on tissue-adhesive-coated glass slides (Fisher Scientific), and stored at -80˚C until use.

### Confocal microscopy

Fluorescent images were acquired by a Leica TCS SP2 confocal microscope (Leica Microsystems) with a pinhole size of 1.5 airy units. Optical confocal images were overlaid and processed with Photoshop and analyzed on a computer screen. The boundaries between the taste bud cells and surrounding non-gustatory epithelial cells were determined in the differential interference contrast images.

### Quantification of non-gustatory epithelial cells expressing fluorescent reporter proteins in the dorsal part of the tongue

Unifluorescent areas in and around the intermolar eminence were identified in the horizontal sections, and the numbers of filiform papillae present in the unifluorescent, i.e., monoclonal areas were counted in the sections of the hillside of filiform papillae (95 areas at 3 months, n = 3 mice; 126 areas at 6 months, n = 3; 58 areas at 12 months, n = 3). Brown-Forsythe and Welch one-way ANOVA and Dunnett’s T3 post hoc tests were carried out to examine if the differences in the numbers of filiform papillae found in the monoclonal areas at 3, 6, and 12 months are significant.

### Quantification of taste buds expressing fluorescent reporter proteins

1. **In fungiform papillae and soft palate.** The number of fluorescent proteins expressed in each single taste bud were identified on serial sections (37 taste buds in soft palate, n = 3 mice; 25 taste buds in fungiform papillae, n = 5 mice). Representative optical confocal images are shown in the figures.
2. **In circumvallate papillae.** Taste bud profiles of every 10 sections of circumvallate papillae were examined, and their preceding and following serial sections were examined to determine the number of fluorescent proteins expressed in a taste bud (107 taste buds at 4 months, n = 3 mice; 160 taste buds at 6 months, n = 5 mice; 137 taste buds at 12 months, n = 3 mice). Brown-Forsythe and Welch one-way ANOVA and Dunnett’s T3 post hoc tests were carried out to examine the significant difference between percentages of taste bud profiles relative to all taste buds expressing at least one fluorescent protein at 4, 6, and 12 months after tamoxifen administration.

### Mathematical estimation of long-lived cells in taste buds

The total number of cells in a taste bud that are not replaced for *t* days ($N_{total}^{t}$) can be estimated as the sum of non-replaced cell numbers of individual cell types ($N_{i}^{t}$) using the half-life (*T_i_*) and the initial number of type *i* cells ($N_{i}^{0}$) with the following equation:

$$N_{total}^{t}=\sum_{i}N_{i}^{t}=\sum_{i}N_{i}^{0}\times {\left(\frac{1}{2}\right)}^{\frac{t}{T_{i}}}$$

Taste buds are made up of 50–100 cells [16,17], and the average taste bud in circumvallate papillae is composed of 30% of *Plcb2*^+^ (also referred to as type II) cells, 16% of *Pkd2l1*^+^ (also referred to as type III) cells, and 54% of others [22]. The half-lives of type II and III cells are 8 and 22 days, respectively. Other taste bud cells, the majority of which are putative non-sensory type I cells, are separated into two subpopulations, referred to as Ia and Ib cells in this study, with half-lives of 8 and 24 days, respectively [12]. Based on the above information, we calculated the number of cells in the taste buds of circumvallate papillae, which presumably have not been replaced for 4, 6, and 12 months.

## Results

### Multicolor lineage tracing of *Sox2*^+^ adult stem cells in the non-gustatory epithelium

**Fig 1:**
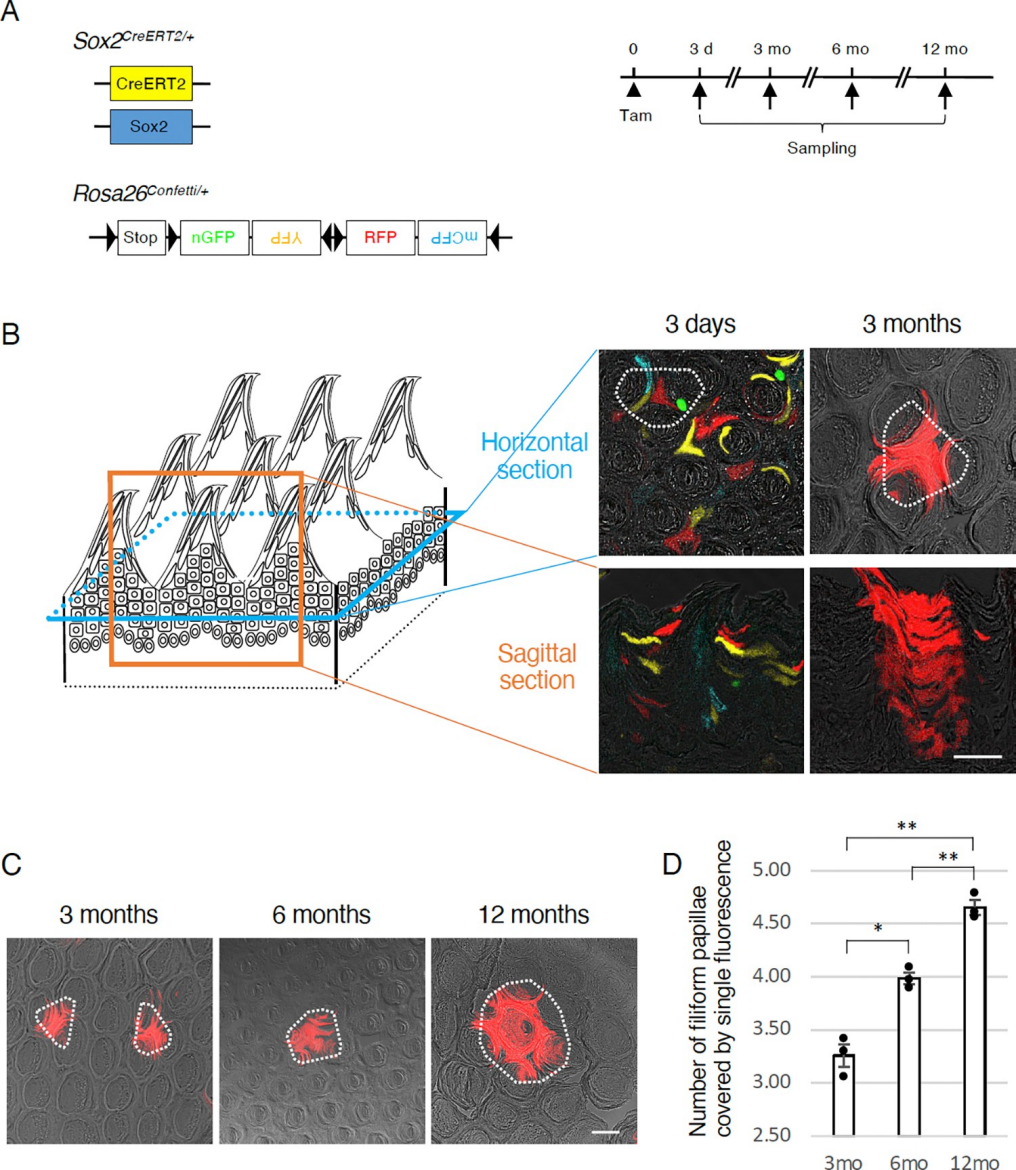
Multicolor lineage tracing of Sox2^+^ stem cells in the filiform papillae. (A) Schematic of multicolor lineage tracing analysis using *Sox2*^*CreERT2*^ [20] and *Rosa26*^*Confetti*^ [19] alleles. Time courses of tamoxifen injection and samplings are shown on the right. (B) Schematic of filiform papillae (FiP, left) and representative confocal images of horizontal (top) and sagittal (bottom) sections of FiP of *Sox2*^*CreERT2/+*^; *Rosa26*^*Confetti/+*^ mice at 3 days and 3 months after a single tamoxifen injection. Fluorescent proteins were observed in the horizontal sections. White dotted lines demarcate the areas of three FiP showing multiple and single fluorescence at 3 days and 3 months, respectively. Green, nGFP (nuclear localization signal-tagged green fluorescent protein); blue, mCFP (membrane-bound type of cyan fluorescent protein); yellow, YFP (yellow fluorescent protein); red, RFP (red fluorescent protein). (C, D) Distribution of cells expressing a fluorescent protein in the FiP at 3, 6, and 12 months after single tamoxifen injection (C). White dotted lines mark the areas of FiP showing single fluorescence. Quantitative analysis of the monoclonal areas exhibiting the same single fluorescence (D). Numbers of FiP included, at least partially, in the monoclonal area were measured (mean ± s.e.m., n = 3), and the differences were statistically examined by one-way ANOVA and Dunnett’s T3 post hoc tests. *p<0.05, **p<0.01. Each data point represents the average number of FiP seen in each mouse. Scale bars, 50 μm.

*Rosa26-Confetti* reporter allele allows the labeling of cells by either none or any of the four fluorescent reporter proteins (nGFP, YFP, RFP, or mCFP) upon Cre-mediated recombination, and all the progenies express the same fluorescent protein as their stem cells, when the recombination is induced in the stem cells. Thus, multicolor labeling and subsequent lineage tracing using *Rosa26-Confetti* reporter allele is very useful for dissecting a group of cells into individual cells and observing the contributions of individual stem cells in the turnover of a tissue of interest. We generated *Sox2*-*Confetti* mice and carried out multicolor lineage tracing to observe the long-term dynamics of epithelial cell turnover maintained by *Sox2*^+^ stem cells (Fig 1A).

In *Sox2-Confetti* mice, 3 days after a single injection of tamoxifen, fluorescently labeled cells were found in the non-gustatory filiform papillae composing stratified keratinized epithelial cells in the dorsal part of the tongue (Fig 1B). A variety of cells expressing different fluorescent reporters and some without any fluorescent reporter, intermingled with each other. Intriguingly, 3 months after tamoxifen injection, small unicolored areas were seen sporadically distributed, where a cluster of cells from basal to apical epithelium express the same reporter protein (Fig 1B). This result suggests that they are derived from the same stem cells. Fluorescently labeled cells were predominantly observed in the inter-papillary regions at 3 months (Fig 1B), suggesting that *Sox2*^+^*Bmi1*^*+*^*Krt5/14*^+^ non-gustatory epithelial stem cells reside not at the base of papillae but at the base of inter-papillary regions, consistent with a previous study [9].

### *Sox2*^+^ adult stem cell dynamics in the non-gustatory epithelium

A previous report had shown that the number of cells within the cluster derived from single *Bmi1*^+^ stem cells in filiform papillae increased after tamoxifen injection, but only till 6 weeks [9]. However, quantitative clonal analysis of *Bmi1*^+^ stem cells showed that the number of labeled epithelial cells per clone increased over time [10]. To determine whether the clonal expansion of *Sox2*^+^*Bmi1*^*+*^*Krt5/14*^+^ stem cells takes place over time, we compared the number of filiform papillae contributing to a unicolored cluster, at least in part, in *Sox2*-*Confetti* mice at 3, 6, and 12 months after tamoxifen injection (Fig 1C and 1D). The number of filiform papillae included at least partially in a unicolored area increased significantly over time (3.26 ± 0.11 (mean ± s.e.m., n = 3) at 3 months, 3.99 ± 0.06 (n = 3) at 6 months, and 4.65 ± 0.07 (n = 3) at 12 months after tamoxifen injection) (Fig 1C and 1D, S1 Table). These results indicate clonal expansion of *Sox2*^+^*Bmi1*^*+*^*Krt5/14*^+^ stem cells over time, implying that among *Sox2*^+^ stem cells in the filiform papillae, some disappear and the others expand, underpinning the turnover of *Sox2*^+^*Bmi1*^*+*^*Krt5/14*^+^ stem cells, and support the population asymmetric division model with neutral competition, as demonstrated in a previous study [10].

**Fig 2:**
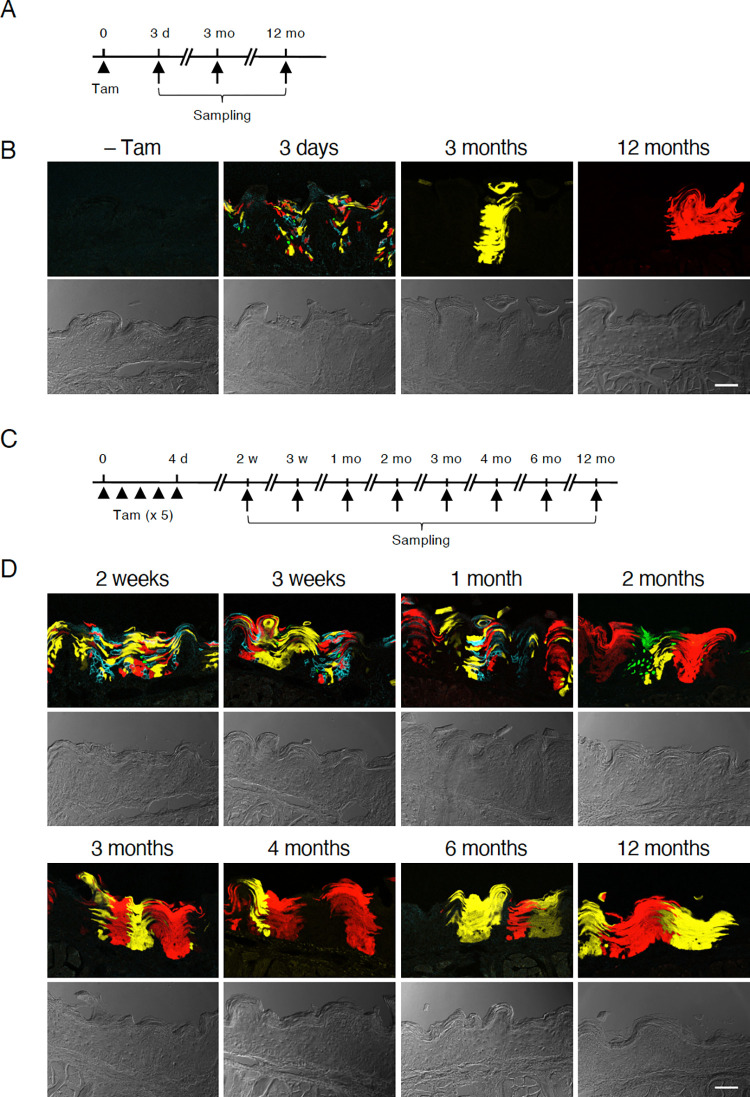
Multicolor lineage tracing of Sox2^+^ stem cells in the non-gustatory epithelium. (A) Time courses of single tamoxifen injection and samplings. (B) Representative fluorescent (top) and bright-field (bottom) images of non-gustatory epithelium in mice without tamoxifen and at 3 days, 3 months, and 12 months after single tamoxifen injection. Without tamoxifen, no fluorescent protein was observed. Green, nGFP (nuclear localization signal-tagged green fluorescent protein); blue, mCFP (membrane-bound type of cyan fluorescent protein); yellow, YFP (yellow fluorescent protein); red, RFP (red fluorescent protein). (C) Time courses of 5 tamoxifen injections and samplings. (D) Representative fluorescent (top) and bright-field (bottom) images of non-gustatory epithelium in mice after tamoxifen injections for 5 consecutive days. Scale bars, 50 μm.

We observed rare clustered cells exhibiting fluorescent reporter protein expression at 3 months or longer after a single tamoxifen injection (Fig 1B and 1C). One possible explanation for the sporadic expression of fluorescent reporter proteins, seen in the long chase for lineage tracing, is that the frequency of reporter-expressing stem cells is so low, that the reporter-negative stem cells dominate through their turnover. We then asked whether tamoxifen injection for multiple days could increase the population of reporter-expressing stem cells in the tongue epithelium. In the non-gustatory, non-papillary epithelium in the most posterior part of the dorsal tongue, the long-term chase of a single tamoxifen injection yielded sporadic unicolored areas that showed columnar structures made of single colored cell clusters in the coronal sections (Fig 2A and 2B), similar to the filiform papillae (Fig 1B). However, tamoxifen injections for 5 consecutive days yielded qualitatively more unicolored columnar areas at 3 months and later (Fig 2C and 2D), suggesting that tamoxifen injections for 5 consecutive days allow us to distinguish the contribution of single stem cells to oral epithelial cell turnover by multicolor tracing. Interestingly, fluorescent reporter expression was mosaic along the basal to apical axis in the short-term chases for a few weeks and did not show clear unicolored columnar areas seen in the 1-month chase (Fig 2D), which turned thicker over time, reminiscent of horizontal growth of unicolored areas in the filiform papillae (Fig 1C and 1D).

### Long-term chase in multicolor lineage tracing of *Sox2*^+^ adult stem cells in the gustatory epithelium

**Fig 3:**
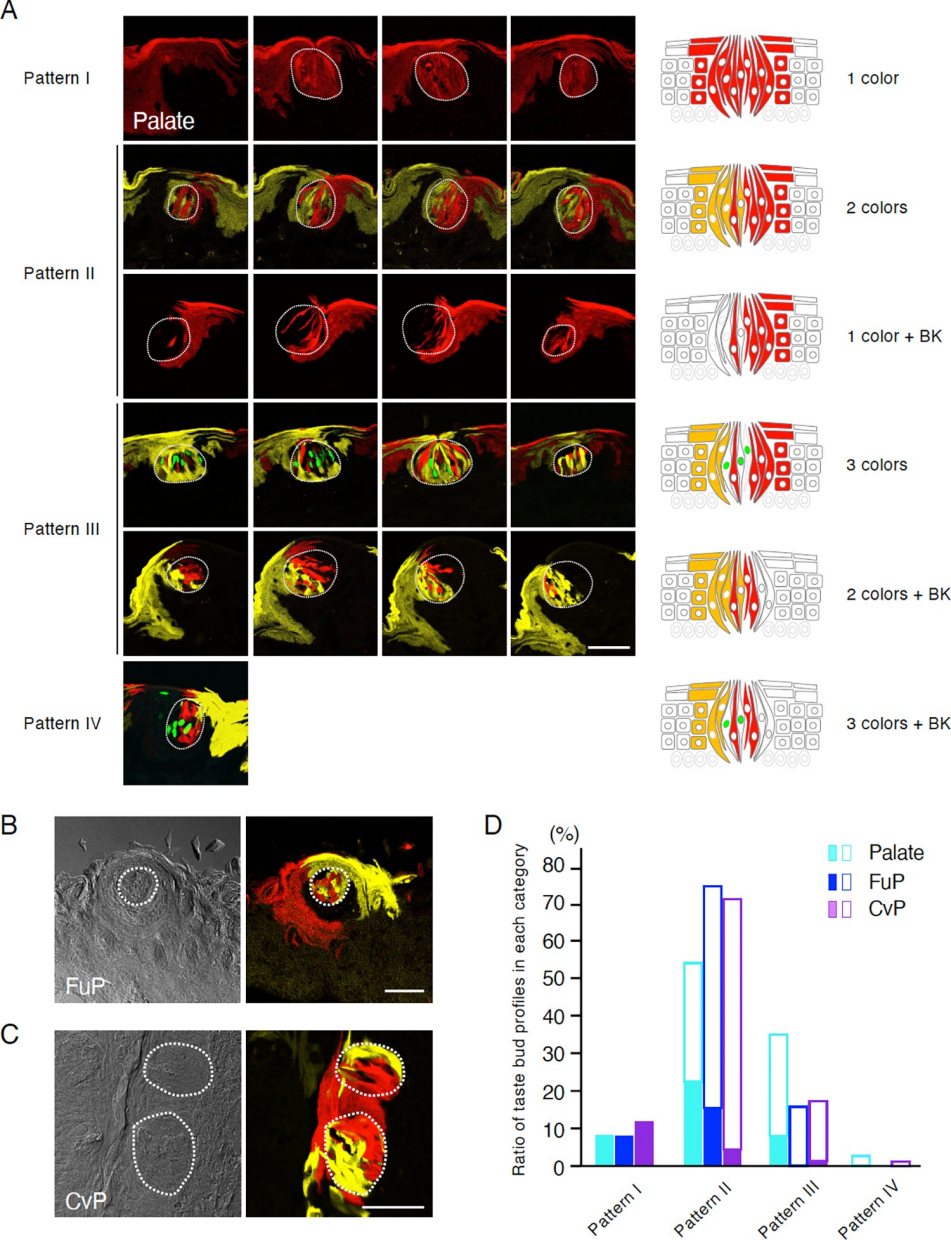
Multicolor lineage tracing of Sox2^+^ stem cells in the epithelium including taste buds. Long-term chase of multicolor lineage tracing was carried out to observe the expression patterns of fluorescent proteins in the taste buds of *Sox2*^*CreERT2/+*^; *Rosa26*^*Confetti/+*^ mice at 6 months after tamoxifen injections for 5 consecutive days. (A) Representative confocal images of fluorescent patterns observed in the taste buds from the soft palate. Serial sections of the epithelium including the same taste bud are shown. Taste buds are outlined by white dotted lines. Taste bud profiles were categorized by the patterns of fluorescence in a single taste bud: Pattern I, single taste buds fully labeled with one fluorescent protein; pattern II, single taste buds labeled fully with two fluorescent proteins or partially with one fluorescent protein; pattern III, single taste buds labeled fully with three fluorescent proteins or partially with two fluorescent proteins; pattern IV, single taste buds labeled partially with three or four fluorescent proteins. (B, C) Representative bright-field (left) and fluorescent (right) images of the taste buds of fungiform papillae (FuP, B) and circumvallate papillae (CvP, C) exhibiting two fluorescence colors in single taste buds. Taste buds are outlined by white dotted lines. Scale bars, 50 μm. (D) Quantitative analysis of expression patterns of fluorescent reporter proteins in taste buds from soft palate (light blue), FuP (dark blue), and CvP (purple). Data are percentages of taste buds fully filled (filled solid columns) or partially filled with fluorescent protein(s) (including non-fluorescent cells; open columns) relative to total taste buds showing fluorescence at least in part (y-axis).

Next, we analyzed the contribution of single stem cells to the gustatory epithelium. Although *Sox2* is also expressed in a subset of taste bud cells [3,23,24], these cells are not involved in the generation of epithelial cells inside or outside the taste buds [3], and taste bud cells are continuously replaced by new cells from stem cells every few weeks. Considering the composition of taste bud cells and their half-lives, we simulated the number of cells remaining after several months in a taste bud in circumvallate papillae and found that >98% of cells were replaced by 4 months (S2 Table). Therefore, temporarily labeled taste bud cells in *Sox2*-*Confetti* mice shortly after tamoxifen injection do not complicate the results of long-term analyses of *Sox2*^+^*Lgr5-6*^*+*^*Krt5/14*^+^ stem cells. In *Sox2*-*Confetti* mice, several months after a single tamoxifen injection, taste buds containing cells that express a fluorescent reporter were rarely observed in any gustatory areas (data not shown). Six months after the multiple tamoxifen injections, however, many taste buds in the soft palate, fungiform papillae, and circumvallate papillae still contained cells that expressed the fluorescent reporter proteins (Fig 3A–3C). We categorized taste bud profiles into four patterns, based on the number of fluorescent colors in a whole taste bud and quantified their frequencies as follows: pattern I for all one color (not including only black, which represents no fluorescence), pattern II for dual colors (either two fluorescence, or one fluorescence and black), pattern III for triple colors (three fluorescence, or two fluorescence and black), and pattern IV for quadruple colors (four fluorescence, or three fluorescence and black) (Figs 3A and S1). In the soft palate, fungiform papillae, and circumvallate papillae, about 90% of the reporter-positive taste buds were multicolored, indicating that single taste buds are composed of cells derived from multiple stem cells (Fig 3). Taste buds and juxtaposed non-gustatory epithelial cells we examined always exhibited the same fluorescent colors. It is very unlikely that taste buds and neighboring non-gustatory epithelial cells surrounding taste buds are derived from distinct stem cells that express the same fluorescent reporter, and therefore, it is most likely that both taste bud cells and the neighboring non-gustatory epithelial cells originate from the same single stem cell, suggesting that individual *Sox2*^+^*Lgr5-6*^*+*^*Krt5/14*^+^ stem cells are bipotent and can give rise to both taste bud cells and neighboring non-gustatory epithelial cells *in vivo*.

### Dynamics of *Sox2*^+^ adult stem cells in the gustatory epithelium

**Fig 4:**
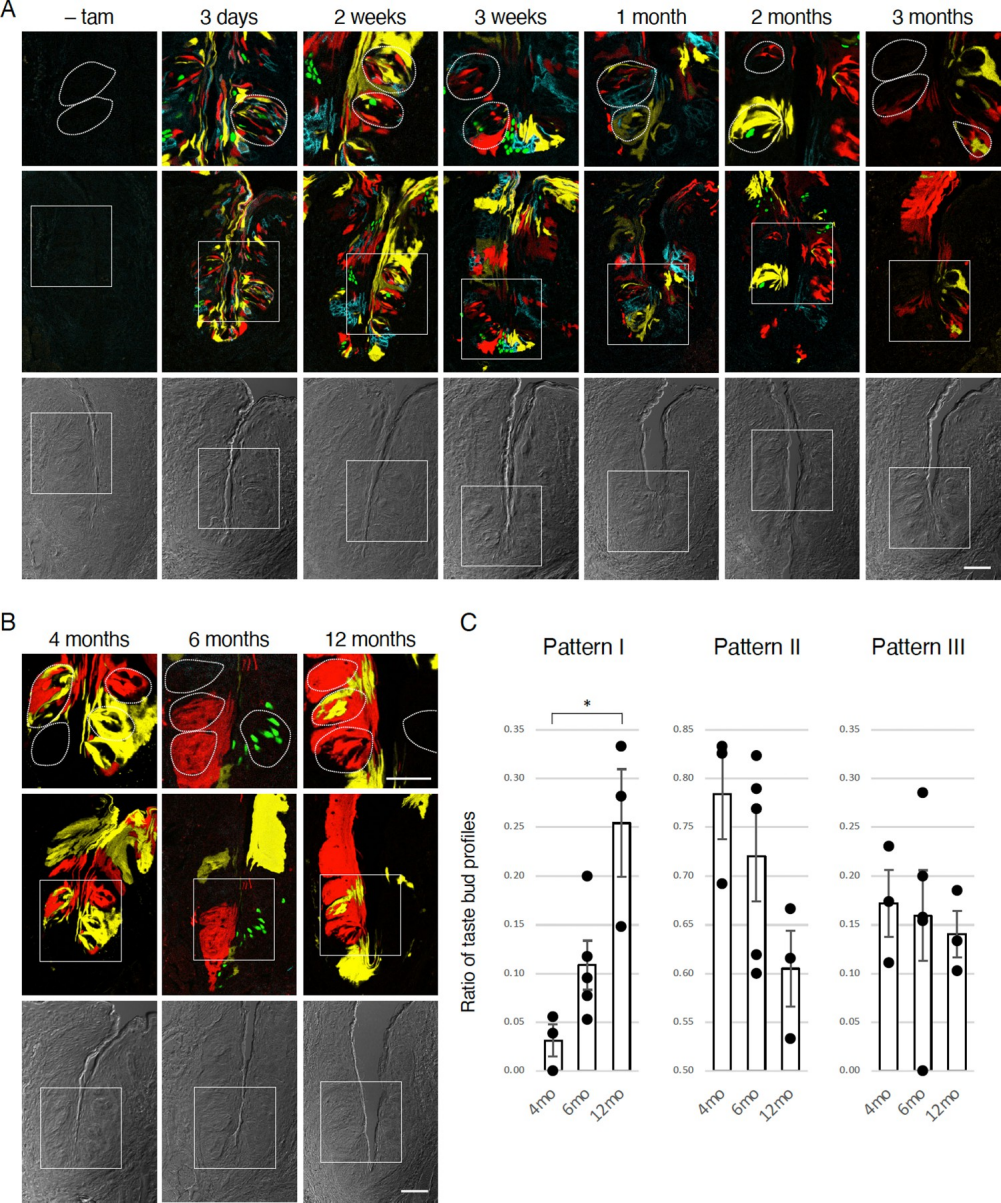
Clonal expansion of single Sox2^+^ stem cell in the gustatory epithelium. (A) Expression of fluorescent proteins in circumvallate papillae (CvP) of *Sox2*^*CreERT2/+*^; *Rosa26*^*Confetti/+*^ mice was examined before tamoxifen injection, at 3 days after single tamoxifen injection, and at up to 3 months after the tamoxifen injections for 5 consecutive days. Magnified (top) and low-power (middle) fluorescent and bright-field (bottom) images of the trench wall of CvP are shown. Areas of magnified fluorescent images are indicated by white squares. Without tamoxifen, no fluorescent protein was observed. (B) Multicolor lineage tracing in CvP of *Sox2*^*CreERT2/+*^; *Rosa26*^*Confetti/+*^ mice at 4, 6, and 12 months after tamoxifen injections for 5 consecutive days. (C) Percentages of single-fluorescence taste buds (pattern I), single taste buds labeled with two fluorescent proteins or with one fluorescent protein and non-fluorescence (pattern II), or single taste buds labeled with three fluorescent proteins or two fluorescent proteins and non-fluorescence (pattern III) relative to all taste buds that expressed at least one fluorescent protein (mean ± s.e.m., n = 3–5). Although one-way ANOVA did not detect significant difference (S3 Table), Welch’s t-test showed significant difference between 4 and 12 months (p = 0.0465). *p<0.05. Each data point represents the data from one mouse. Scale bars, 50 μm.

Lastly, we examined whether the clonal expansion of *Sox2*^+^*Lgr5-6*^*+*^*Krt5/14*^+^ stem cells in the gustatory epithelium is similar to that of *Sox2*^+^*Bmi1*^*+*^*Krt5/14*^+^ stem cells in the filiform papillae, by analyzing the changes in the distribution of the cells that are positive for fluorescent reporters over time in the circumvallate papillae. Epithelial cells, including taste bud cells, had highly mosaic patterns, with different fluorescent reporter proteins in short-term chases (≤1 month), which are not long enough to replace all of the *Sox2*^+^ taste bud cells with new ones. In long-term chases (≥ 3 months after tamoxifen injection), unicolored areas comprising a cluster of cells expressing the same fluorescent protein, were seen more frequently than short-term chases (Fig 4), similar to the observation in non-gustatory epithelium (Figs 1 and 2). Considering that the average life span of taste bud cells is estimated to be a few weeks [11,12], 4 months are long enough to replace many cells (>98%, see S2 Table) in a taste bud at least once, and thus *Sox2*^+^ taste bud cells that were induced to express a fluorescent reporter are not likely to exist after 4 months post-injection. Thus, fluorescently labeled cells in long-term chases most probably originated from stem cells outside the taste buds. The ratio of the taste buds fully labeled with a single fluorescent protein (categorized into pattern I) to whole taste buds in which at least one fluorescent reporter protein was expressed in some cells was 3.1 ± 1.64% (mean ± s.e.m., n = 3) at 4 months and increased to 10.8 ± 2.52% (n = 5) at 6 months and 25.5 ± 5.52% (n = 3) at 12 months (Fig 4B and 4C, S3 Table). These results strongly suggest that, as a part of turnover of epithelial cells in the tongue including taste bud cells, some of the *Sox2*^+^*Lgr5-6*^*+*^*Krt5/14*^+^ stem cells are actively replaced by the other *Sox2*^+^*Lgr5-6*^*+*^*Krt5/14*^+^ stem cells, as explained by the neutral competition model.

## Discussion

In this multicolor lineage tracing study, we showed that cells in a single taste bud are supplied from multiple *Sox2*^+^*Lgr5-6*^*+*^*Krt5/14*^+^ stem cells. We also observed increases of unicolored taste buds over time and unicolored areas in both the non-gustatory and gustatory regions of the tongue. It is likely that *Sox2*^+^*Lgr5-6*^*+*^*Krt5/14*^+^ stem cells are maintained by turnover and not all stem cells are long-lived, as explained by the population asymmetric division model with neutral drift.

### Maintenance model of *Sox2*^+^ gustatory stem cells in the oral epithelium

For tissues maintained by continuous turnover, such as epithelial cells in the alimentary tract including the oral cavity, the maintenance of local resident stem cells is very critical. Two major models have been proposed to explain how the stem cells are maintained [25,26]. One is the neutral drift model, in which some stem cell populations disappear and the others increase and compensate this loss. The other is the invariant asymmetric division model, where a stem cell generates all cells including itself, transit-amplifying cells, and differentiating and differentiated cells in a limited (i.e., clonal) area stably. The increase in unicolored taste buds over time in our long-term multicolor lineage tracing revealed that *Sox2*^+^*Lgr5-6*^*+*^*Krt5/14*^+^ gustatory stem cells are also maintained by the loss of some cells and the promotion of others among them (Figs 1D and 4C), reminiscent of the dynamics of *Lgr5*^+^ stem cells in the intestine, explained by the neutral drift model [18,19]. However, about 75% of the reporter-positive taste buds were still multicolored at 12 months after the induction of recombination (Fig 4C). One possible implication is that many *Sox2*^+^*Lgr5-6*^*+*^*Krt5/14*^+^ stem cells are long-lived, and thus, neutral competition occurs slowly. It is also possible that some *Sox2*^+^ cells are slow-cycling stem cells [27], and generate taste bud cells. In addition, we cannot preclude the possibility of extremely long-lived taste bud cells. It would be interesting to characterize slow-cycling stem cells and perform pulse-chase studies of taste bud cells to determine whether there are extremely long-lived cells in the taste buds.

### Maintenance model of *Sox2*^+^ non-gustatory stem cells in the oral epithelium

Two models have been proposed for *Bmi1*^+^ non-gustatory stem cell maintenance in the oral epithelium. *Bmi1*^+^ stem cells are (1) slow-cycling cells maintained by the invariant asymmetric divisions [9] and (2) rapid-cycling cells maintained by the population asymmetric division model with neutral drift [10]. Jones et al. also demonstrated the absence of slow-cycling stem cells in the oral epithelium and that *Bmi1*^+^ cells express *Sox2* [10]. In the filiform papillae of *Sox2*-*Confetti* mice several months after a single tamoxifen injection, fluorescent reporter-positive areas expanded and were unicolored. Thus, it is unlikely that slow-cycling stem cells, if any, contributed to the generation of non-gustatory epithelial cells in the tongue. Therefore, our results using *Sox2-Confetti* mice are consistent with and support the previous study by Jones et al. [10] with regard to the following three points: (1) *Sox2*^+^*Bmi1*^*+*^*Krt5/14*^+^ stem cells are maintained by population asymmetric divisions and neutral drift, (2) *Sox2*^+^*Bmi1*^*+*^*Krt5/14*^+^ stem cells are not slow-cycling cells, and (3) the ordinary turnover of non-gustatory epithelial cells does not involve slow-cycling cells [10].

### Multipotency of *Sox2*^+^ adult gustatory stem cells for epithelial lineages

Taste buds contain non-sensory cells and multiple types of (i.e., sweet, umami, bitter, sour, and salty) taste cells. Unicolored taste buds increased over time, albeit slowly, suggesting that all types of cells in a taste bud originate from one stem cell, as observed in *in vitro* cultured taste organoids derived from single *Lgr5*^+^ and *Lgr6*^+^ stem cells [8]. However, we cannot completely preclude the possibility that cells in unicolored taste buds are derived from multiple stem cells expressing the same fluorescent protein. It is still unclear whether each stem cell generates all types of taste bud cells (i.e., each stem cell is equally multipotent) or only specific types of stem cells do (i.e., individual stem cells have some differences in their potency) *in vivo*. Short-term lineage tracing of only a small population of stem cells (e.g., *Krt5*^*CreERT2/+*^*; Rosa26*^*Confetti/+*^ mice or *Krt5*^*CreERT2/+*^*; Rosa26*^*tdTomato/+*^ with low doses of tamoxifen) may provide more robust evidence to resolve this matter.

### Nature of *Sox2*^+^ gustatory stem cells

Taste buds show regional differences in their molecular features, represented by the composition of taste cells [4,28–33], and epithelial stem cells in the tongue also show regionally different features; e.g., *Lgr5* expression is restricted to circumvallate and foliate papillae present in the posterior part of the tongue in adult mice [6,7]. It would be interesting to examine whether the gustatory stem cells are intrinsically homogeneous, with extrinsic factor(s) capable of differentially altering their fates, or they are innately and regionally different and hence, generate different taste cell subsets in single taste buds. Transcriptomic analyses of single stem cells may provide answers or new insights into the nature of gustatory stem cells.

## Conclusion

Maintenance of stem cells is pivotal for tissue homeostasis of the oral epithelium, including non-gustatory and taste bud cells. Present study revealed that multiple stem cells contributed to a single taste bud. Further, not all stem cells were long-lived; some of them were lost, while the others increased and compensated for the loss. These findings provide new insights into the turnover of stem cells and epithelial cell lineages.

## Supporting information

S1 Fig Other examples of taste buds in the soft palate at 6 months after tamoxifen injections. Multicolor lineage tracing was carried out to observe the expression patterns of fluorescent proteins in the taste buds of *Sox2*^*CreERT2/+*^; *Rosa26*^*Confetti/+*^ mice at 6 months after tamoxifen injections for 5 consecutive days. Confocal images of fluorescent patterns observed in the taste buds of the soft palate are shown. Taste buds are outlined by white dotted lines. Taste bud profiles were categorized by fluorescence patterns in a single taste bud: Pattern I, single taste buds fully labeled with one fluorescence; pattern II, single taste buds labeled fully with two fluorescence or partially with one; pattern III, single taste buds labeled fully with three fluorescence or partially with two. Scale bar, 50 μm.
(TIF)

S1 Table Summary of statistical analyses of monoclonal areas in FuP. (DOCX)

S2 Table Estimation of the number of cells that are not replaced in a taste bud in circumvallate papillae during certain periods of time (*t* days). (DOCX)

S3 Table Summary of statistical analyses of ratio of single-fluorescence taste buds. (DOCX)
