# Supplementary figures and images for: Maintenance and turnover of Sox2+ adult stem cells in the gustatory epithelium
Source: PLoS One. 2022 Sep 2;17(9):e0267683. doi: 10.1371/journal.pone.0267683 (PMC9439239; doi:10.1371/journal.pone.0267683)

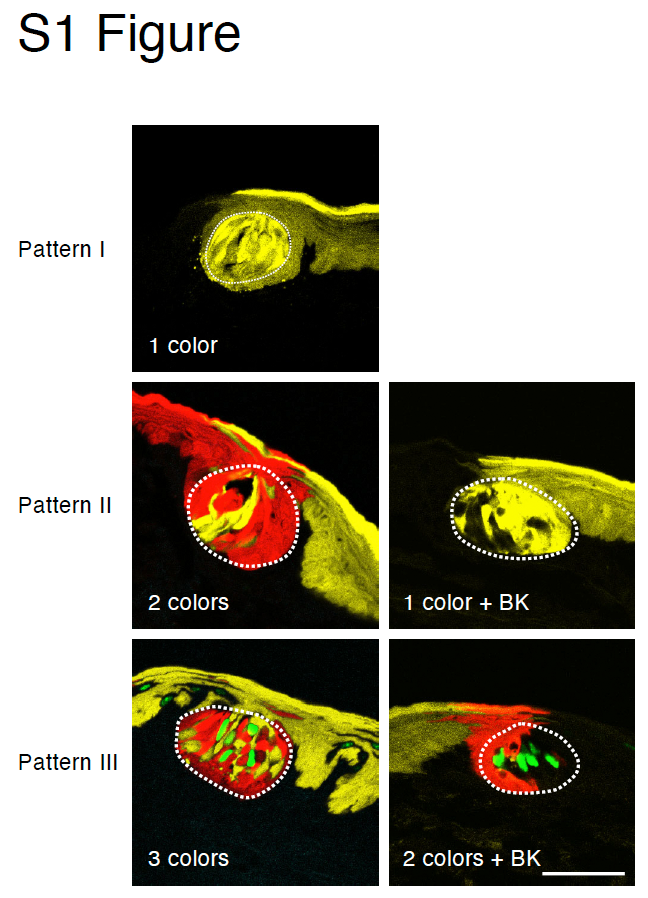

Supplement: S1 Fig — Multicolor lineage tracing was carried out to observe the expression patterns of fluorescent proteins in the taste buds of Sox2CreERT2/+; Rosa26Confetti/+ mice at 6 months after tamoxifen injections for 5 consecutive days. Confocal images of fluorescent patterns observed in the taste buds of the soft palate are shown. Taste buds are outlined by white dotted lines. Taste bud profiles were categorized by fluorescence patterns in a single taste bud: Pattern I, single taste buds fully labeled with one fluorescence; pattern II, single taste buds labeled fully with two fluorescence or partially with one; pattern III, single taste buds labeled fully with three fluorescence or partially with two. Scale bar, 50 μm. (TIF) [file pone.0267683.s001.tif]
