# Supplementary material for: Maintenance and turnover of Sox2+ adult stem cells in the gustatory epithelium
Source: PLoS One. 2022 Sep 2;17(9):e0267683. doi: 10.1371/journal.pone.0267683 (PMC9439239; doi:10.1371/journal.pone.0267683)
Supplement: S1 Table — (DOCX) [file pone.0267683.s002.docx]

| S1 Table. Summary of statistical analyses of monoclonal areas in FuP | | |
| --- | --- | --- |
|  |  |  |
|  | Welch-corrected t, df | P value |
| 3 mo vs 6 mo | t=6.058, df=3.09 | *P*=0.0213 |
| 3 mo vs 12 mo | t=11.06, df=3.456 | *P*=0.0038 |
| 6 mo vs 12 mo | t=7.43, df=3.866 | *P*=0.0045 |
