# Supplementary material for: Maintenance and turnover of Sox2+ adult stem cells in the gustatory epithelium
Source: PLoS One. 2022 Sep 2;17(9):e0267683. doi: 10.1371/journal.pone.0267683 (PMC9439239; doi:10.1371/journal.pone.0267683)
Supplement: S2 Table — (DOCX) [file pone.0267683.s003.docx]

| S2 Table. Estimation of the number of cells that are not replaced in a taste bud in circumvallate papillae during certain periods of time (*t* days). | | | | | |
| --- | --- | --- | --- | --- | --- |
|  |  |  |  |  |  |
| Cell type | half-life^*1^ (days) | Number of non-replaced cells | | | |
|  |  | *t* = 0 | *t* = 120 | *t* = 180 | *t* = 360 |
| Ia | 8 | 27 | 0.00 | 0.00 | 0.00 |
| Ib | 24 | 27 | 0.84 | 0.15 | 0.00 |
| II | 8 | 30 | 0.00 | 0.00 | 0.00 |
| III | 22 | 16 | 0.36 | 0.06 | 0.00 |
| total | n/a^*2^ | 100 | 1.21 | 0.20 | 0.00 |

^*1^ [12]

^*2^ not applicable
