# Supplementary material for: Maintenance and turnover of Sox2+ adult stem cells in the gustatory epithelium
Source: PLoS One. 2022 Sep 2;17(9):e0267683. doi: 10.1371/journal.pone.0267683 (PMC9439239; doi:10.1371/journal.pone.0267683)
Supplement: S3 Table — (DOCX) [file pone.0267683.s004.docx]

| S3 Table. Summary of statistical analyses of ratio of single-fluorescence taste buds | | |
| --- | --- | --- |
|  |  |  |
|  | Welch-corrected t, df | P value |
| 3 mo vs 6 mo | t=2.561, df=5.965 | *P*=0.1109 |
| 3 mo vs 12 mo | t=3.875, df=2.351 | *P*=0.1246 |
| 6 mo vs 12 mo | t=2.406, df=2.862 | *P*=0.2121 |
